# Maternal Exposure to Low Levels of Corticosterone during Lactation Protects against Experimental Inflammatory Colitis-Induced Damage in Adult Rat Offspring

**DOI:** 10.1371/journal.pone.0113389

**Published:** 2014-11-18

**Authors:** Carla Petrella, Chiara Giuli, Simona Agostini, Valérie Bacquie, Manuela Zinni, Vassilia Theodorou, Maria Broccardo, Paola Casolini, Giovanna Improta

**Affiliations:** 1 Department of Physiology and Pharmacology “V. Erspamer”, Sapienza University of Rome, Rome, Italy; 2 INRA, EI-Purpan, UMR 1331 TOXALIM Neuro-Gastroenterology and Nutrition Team, Toulouse, France; Duke University Medical Center, United States of America

## Abstract

Opposing emotional events (negative/trauma or positive/maternal care) during the postnatal period may differentially influence vulnerability to the effects of stress later in life. The development and course of intestinal disorders such as inflammatory bowel disease are negatively affected by persistent stress, but to date the role of positive life events on these pathologies has been entirely unknown. In the present study, the effect of early life beneficial experiences in the development of intestinal dysfunctions, where inflammation and stress stimuli play a primary role, was investigated. As a “positive” experimental model we used adult male rat progeny nursed by mothers whose drinking water was supplemented with moderate doses of corticosterone (CORT) (0.2 mg/ml) during the lactation period. Such animals have been generally shown to cope better with different environmental situations during life. The susceptibility to inflammatory experimental colitis induced by intracolonic infusion of TNBS (2,4,6-trinitrobenzenesulphonic acid) was investigated in CORT-nursed rats in comparison with control rats. This mild increase in maternal corticosterone during lactation induced, in CORT-nursed rats, a long lasting protective effect on TNBS-colitis, characterized by improvements in some indices of the disease (increased colonic myeloperoxidase activity, loss of body weight and food intake) and by the involvement of endogenous peripheral pathways known to participate in intestinal disorder development (lower plasma corticosterone levels and colonic mast cell degranulation, alterations in the colonic expression of both corticotrophin releasing factor/CRF and its receptor/CRH-1R). All these findings contribute to suggesting that the reduced vulnerability to TNBS-colitis in CORT-nursed rats is due to recovery from the colonic mucosal barrier dysfunction. Such long lasting changes induced by mild hormonal manipulation during lactation, making the adult also better adapted to colonic inflammatory stress, constitute a useful experimental model to investigate the etiopathogenetic mechanisms and therapeutic treatments of some gastrointestinal diseases.

## Introduction

Early life experiences profoundly influence the later development, the structure and function of an organism.This phenomenon, called “developmental programming,” is a process whereby an environmental factor acting during a sensitive or vulnerable developmental period exerts effects that, in some cases, will persist throughout life.

Adaptive or maladaptive responses to environmental stressors reflect an animal's capacity to re-establish temporarily disrupted physiological homeostasis. A number of factors contribute to the qualitative nature of these responses such as: the intensity (high or low) and duration (acute or chronic) of stressors, the individual's ability to initiate an adaptive response, and the phase of the life when the stressor event occurs. In particular, concerning the latter point, during postnatal life, a critical period for neuroendocrinological and behavioural development processes, different emotional events (negative/trauma or positive/maternal care) may influence, in opposite ways, vulnerability to the effects of stress later in life, possibly by inducing a persistent sensitization in stress-responsive neural circuits [1]–[5].

“Neonatal maternal deprivation” is one of the best known experimental animal models that well reproduces in rodents the consequence of traumatic experiences occurring in humans in early life. In particular, the stress evoked by altering mother–infant interactions during lactation causes the offspring, once adult, to develop a phenotype more susceptible to stress events and characterized by hyperactivation of the Hypothalamus – Pituitary – Adrenal (HPA) axis. Interestingly, the pathophysiological modifications observed in adult rats affect not only the behaviour and the neuroendocrine system, but also the homeostasis of the gastrointestinal tract. In fact, adult rats separated early postnatally from their mothers have been found to be predisposed to colonic barrier dysfunction [6], [7] and to have an enhanced mucosal response to stress [8]. These findings are in line with evidence that shows that adverse experiences early in life can have implications in the development and the clinical course of human intestinal disorders, including inflammatory bowel disease (IBD) and intestinal bowel syndrome (IBS) [9]–[11], where inflammatory and stress stimuli play primary roles [12], [13].

On the other hand, experiences, during human infancy, involving dynamic, tender, and stimulating environments, may have positive long lasting effects on the quality of life, can serve as a source of resilience in the face of chronic stress [14], [15], and tend to promote resistance to stress and diminish vulnerability to stress-induced illness [16], [17]. In recent years, several experimental animal models have well represented this evidence. Environmental enrichment has been used as a procedure that might prevent some of the deleterious effects of stress [18], [19]. Francis et al. [20] have furthermore demonstrated that environmental enrichment can reverse the effects of postnatal maternal separation on both endocrine and behavioural responses to stress. Moreover, the offspring of mothers displaying high levels of maternal care (such as licking and grooming), once adults, increased their exploratory behaviour and spatial memory and reduced their anxiety-like behaviour [21], [22]. In our previous studies [23] conducted in rats, we have shown that offspring nursed by mothers with a mild hypercorticosteronemia (that reflects a form of mild environmental stimulation) develop the ability to cope better with different situations during life. In this animal model, the drinking water of mother rats during lactation was supplemented with corticosterone (0.2 mg/ml) [24], [25]. Maternal corticosterone is in equilibrium between blood and milk in rodents [23]–[25], and the hormone is easily absorbed by the gastrointestinal tract of the pups, as the glucocorticoid permeability of the gut is very high in early postnatal life up to 17–18 days of age [25]. With this approach a moderate increase in corticosterone (mimicking mild stimulating stress) may be achieved in the mother as well as in the pups without disturbing them. The progeny of these mothers (CORT-nursed rats), once adults, showed improved learning capabilities, reduced fearfulness in anxiogenic situations and, more interestingly, resistance to ischemic neuronal damage [11], [23]. The protective long-life effect of hormonal manipulation in CORT-nursed rats is strictly linked to a persistent hyporeactivity of the HPA axis due to an increased number of glucocorticoid receptors in the hippocampus, a recognized target of glucocorticoid negative feedback action [26], [27].

To our knowledge, there have been no studies considering the effect of such a positive postnatal manipulation on the homeostasis of the gastrointestinal tract. Therefore, the aim of the present study was to investigate the susceptibility to inflammatory colitis induced by intracolonic infusion of TNBS (2,4,6-trinitrobenzenesulphonic acid) in adult CORT-nursed offspring.

The data presented in this work show the long lasting effect of mildly increased maternal corticosterone during lactation on TNBS colitis in three-month old male rats, and take into account the variations in some indices of the pathology (histological score, colonic MPO activity, body weight and food intake) and the involvement of the main peripheral endogenous systems: mast cells, glucocorticoids (GCs) and their receptors (GRs), corticotrophin releasing factor (CRF) and its receptor, CRH-1R, known to be involved in the onset and progression of colitis [10], [28]–[33].

Taken together, these results demonstrate that a mild hormonal manipulation during lactation protects against the onset of TNBS-induced colitis.

## Materials and Methods

### Ethics Statement

All animal procedures were carried out according to EU Directive 86/609/EEC and to Italian legislation on animal experimentation. The experimental protocols were also approved by the Local Animal Care and Use Committee of Institut National de la Recherche Agronomique (authorization number MP/02/46/11/08).

### CORT-nursed model

Female Wistar rats (Charles River, Calco, Italy) weighing 280–320 g were mated and then housed individually. The day after the birth, litters were culled to eight pups (four males and four females). Mothers of control rats were maintained on tap water, whereas mothers of CORT-nursed rats had ad libitum access to a solution of 0.2 mg/ml corticosterone hemisuccinate. Weaning was performed at 21 day of age, and animals were then housed three per cage. Three-month-old male CORT-nursed rats and their controls were used in this study They were kept in a temperature-controlled room (21°C), and were allowed free access to water and food.

### Experimental design

The first day of experiment (day 0), both controls (n = 24) and CORT-nursed (n = 24) animals were divided in two groups of 12 rats each one: 1) healthy rats, intracolonically infused with saline, and 2) colitic rats, intracolonically infused with TNBS (2,4,6-trinitrobenzenesulphonic acid). To avoid the litter effect, each litter contributed one or, maximum, two off spring per group. On the 4^th^ day after TNBS and/or saline instillation all the animals were euthanized by CO_2_ inhalation (till 13 PM) and, for each experimental group, colonic tissue and blood samples were collected.

### Induction of experimental colitis

Overnight fasted control and CORT-nursed rats were anesthetised by intraperitoneal injection of xilazine (0.6 mg/kg) and ketamine (120 mg/kg), and colitis was induced by an intra-colonic (IC) administration of TNBS at a dose of 30 mg/kg in 0.3 mL of 50% ethanol. Healthy rats were intracolonically infused with 0.3 mL of saline. TNBS and/or saline were infused through a silicone catheter introduced in the distal colon, 6 cm into the anus as previously described [34].

### Body weight and food intake

Animal body weight and food intake were registered on the day of IC instillation of TNBS or saline and on the day of sacrifice. The index of variation was expressed as difference between the final and the initial weight (g).

### Histological evaluation

#### Intestinal length

After the sacrifice, entire colons were removed and rinsed with saline. Colonic length was evaluated, measuring the distance from the caecum to the anus.

#### Macroscopic damage

Immediately after sacrifice, colon samples were removed and rinsed with saline. Colonic damage was evaluated in double blind and expressed with a score that took into account the severity and extent of macroscopic lesions (hyperemia, ulcers), the presence and the extent of adhesions and the occurrence of diarrhoea, according to a modified Wallace *et al.* scale [35].

#### Microscopic damage

Samples of distal colon were fixed in Duboscq-Brazil buffer, dehydrated and embedded in paraffin. Sections of 5 µm were stained with eosin-hemalun, and examined by light microscopy. The extent of histological damage was expressed according to the criteria described by Fabia R, *et al* [36]. Each parameter estimated was graded (0–3), depending upon the severity of the changes found: (0) no change, (1) mild, (2) moderated or (3) severe changes. Total damage was obtained by adding the individual scores.

### Myeloperoxidase (MPO) activity assay

The activity of MPO, a marker of polymorphonuclear primary granules, was determined in colon tissue according to a previously described technique [37]. Immediately after sacrifice, a distal colonic segment (1 cm long) was taken off at 3 cm from the ceco-colonic junction. It was suspended in potassium phosphate buffer (KH_2_PO_4_ 44 mM, K_2_HPO_4_ 6 mM, pH 6.0), homogenized on ice with Polytron (PCU-2, Kinematica GmbH, Lucerne, Switzerland) and submitted to three cycles of freezing and thawing. Homogenates were then centrifuged at 9000 *g* for 15 min at 4°C. The pellets were resuspended in hexadecyl trimethylammonium bromide buffer (0.5% (wt/vol) in potassium phosphate buffer) to release MPO from polymorphonuclear neutrophil primary granules. These suspensions were sonicated (Büchi, Flawil, Switzerland) on ice and centrifuged at 9000 *g* for 15 min at 4°C. Supernatant fractions were diluted in potassium phosphate buffer containing 0.167 mg o-dianisidine dihydrochloride/ml and 0.00005% (vol/vol) H_2_O_2_. MPO from human neutrophils (Sigma, Saint Quentin Fallavier, France; 0.1 U/ml) was used as a standard. Changes in absorbance at 450 nm were recorded with a spectrophotometer (mc2UV, Safas, Monaco) every 10 s over 2 min. One unit of MPO activity was defined as the quantity of MPO degrading 1 µmol H_2_O_2_ min^−1^ml^−1^ at 25°C. Protein concentrations (µg/ml) were determined using a modified method of Lowry (Detergent Compatible Assay, BioRad, Ivry/Seine, France) and MPO activity was expressed as MPO units/g protein.

### Corticosterone assay

Samples of blood (5 ml) were collected in tubes containing EDTA (200 µl/5 ml). After centrifugation at 3300×*g* at 4°C for 20 min, plasma was removed and kept frozen at −20°C, till the time of the test. Samples were then processed for ELISA using commercial kits to determine plasma corticosterone concentrations (ELISA kits, Demeditec Diagnostic, Germany). Data were expressed as µg/100 ml.

### Colonic mucosal GR expression

Colonic mucosa samples from control or CORT-nursed rats were stored frozen at −80°C. On the day of the experiment, tissue was sonicated at 4°C in 300 µl of high salt EPG buffer (1 mM EDTA, 20 mM phosphate buffer pH 7.4, 10% glycerol, 0.4 M NaCl, 5 mM dithiothreithol) containing protease inhibitors (phenylmethylsulfonyl fluoride 1 mM, leupeptin 10 µg/ml and aprotinin 10 µg/ml). Protein concentrations were determined using the Bradford protein assay. Thirty micrograms of protein were re-suspended in sodium dodecyl sulfate (SDS)-bromophenol blue loading buffer with 0.7 M 2-mercaptoethanol. The samples were boiled for five minutes and separated on 8% SDS-polyacrylamide gels. After electrophoresis (Protean II xi System, Bio-Rad), the proteins were transferred to nitrocellulose membranes (Biorad) using a system of maxi transblot cell (BioRad) at 4°C. After transfer, blots were incubated in a solution (blocking solution) containing Tris-buffered saline (TBS), 10% (w/v) Tween-20, 1% (w/v) non-fat milk and 1% (w/v) bovine serum albumin. Subsequently, blots were incubated overnight with rabbit anti-GR (1∶10000, sc-1004, Santa Cruz Biotechnology Inc.) in blocking solution at 4°C. After incubation with the primary antibody, the blots were incubated with horseradish peroxidase-conjugated goat anti-rabbit (1∶10000; Amersham Bioscience) for 1 h at room temperature (21°C±2). To ensure that each lane was loaded with an equivalent amount of protein, the blots were probed with an anti-actin serum (1∶1000; Sigma) overnight at 4°C. Subsequently, blots were incubated with horseradish peroxidase-conjugated goat anti-mouse antibodies (1∶5000; Amersham Bioscience) for 1 h at room temperature. Bands were visualized with an enhanced chemiluminescence system (Aurogene). After immunoblotting, digitized images of bands immunoreactive for target (GR) and control (actin) molecules were acquired, and the area of immunoreactivity corresponding to each band was measured, using the NIH ImageJ medical imaging software. A ratio of target to actin was then determined, and these values were compared for statistical significance.

### Colonic CRF and CRH-1R immunostaining

Colon samples were immediately fixed in Dubosq-Brazil solution for 24 h, dehydrated in ethanol solution, embedded in paraffin blocks and cut into 5 mm sections. Paraffin sections were rehydrated and submerged in antigen retrieval solution (citrate buffer, 10 mM, ph 6, 95°C, 3 min). After inhibition of endogenous peroxidases with 0.6% H_2_O_2_ in PBS for 30 min, and incubation in blocking solution (phospate-buffered saline containing 1% bovine serum albumin and 2% normal donkey serum), sections were incubate with goat anti-CRF (sc-21675; 1/100) or goat anti CRH-1R (sc-12383; 1/100) antibodies (Santa Cruz, Le Perray en Yvelines, France), overnight at 4°C. Subsequently, slides were incubated with biotinylated donkey anti-goat IgG immune serum (Interchim, Montluçon, France) 1/1000, for 30 min at room temperature, and added with ABC complexes coupled to peroxidase (Clinisciences, Nanterre, France). 3–3′ diaminobenzidine (Clinisciences) was used as chromogen. CRF immunoreactive area/mm^2^ of mucosa and epithelium, CRH-1R immunoreactive area/mm^2^ of epithelium and CRH-1R. DO intensity in mucosal immune cells were quantified using Nikon-Elements-Ar software (Nikon, Champigny-sur-Marne, France).

### Colonic rat mast cell protease II (RMCP II) immunostaining

Distal colonic samples were collected and fixed in 4% paraformaldehyde and immersed for 24 h in 30% of sucrose at 4°C. Samples were embedded in Neg 50 medium (MM France, Francheville, France) and frozen in isopentane at −45°C. Cryostat sections (7 µm) were post-fixed with acetone (10 min, −20°C) and hydrated in phosphate-buffered saline (PBS). After incubation in blocking solution (PBS-1% BSA-2% normal donkey serum, sections were incubated overnight at 4°C with sheep anti-rat mast cell protease (RMCP) II (1/500) (Moredun, Midlothian, UK) antibody. Sections were washed in PBS and incubated for 1.5 h at room temperature with Alexa fluor 594-conjugated immunoglobulin G (IgG) donkey anti-sheep (1/2000) antibody (Life technologies, Paris, France). Sections were mounted in Prolong gold antifade mounting medium (Life Technologies) and examined under a Nikon 90i fluorescence microscope (Nikon). Number of mast cells per mm^2^ and the immunoreactive area/mm^2^ of mucosa were quantified using Nikon-Elements-Ar software.

### Statistical analysis

All data are presented as means ± SEM. For statistical analysis Graph Pad Prism 4.0 (GraphPad, San Diego, CA) was used. Results were analysed by one way ANOVA followed by Dunnett's Multiple Comparison Test. A. Statistical significance was set at p<0.05.

## Results

### Body weight and food intake

**Figure 1:**
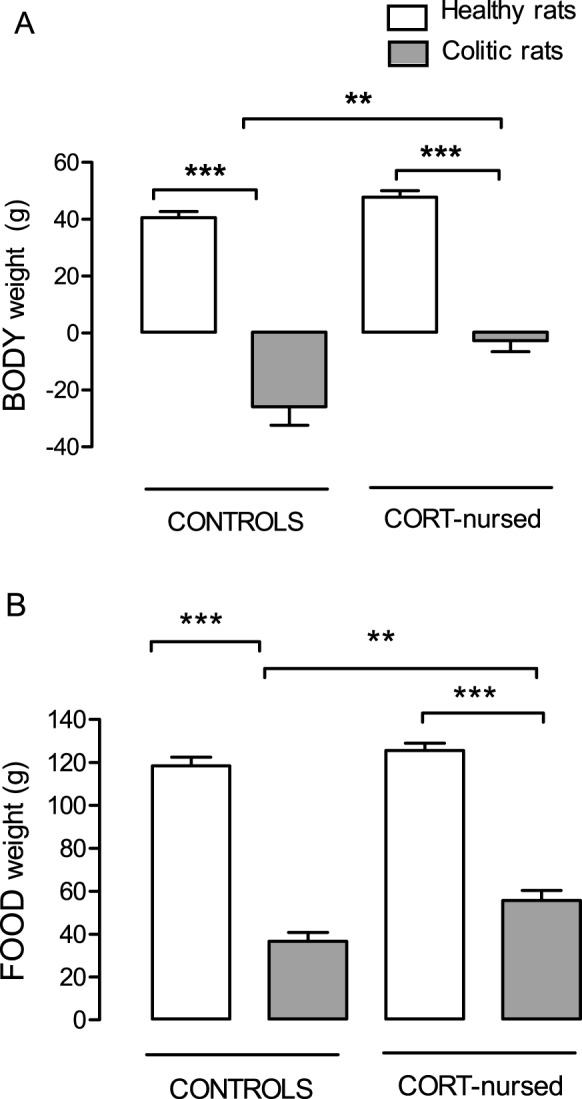
Effect of TNBS-colitis on body weight and food intake in control and CORT-nursed rats. Induction of colitis reduced body weight (**A**) and food intake (**B**) both in control and CORT-nursed rats. The decrease in both parameters in colitic CORT-nursed rats was significantly less than in colitic controls. **p<0.01; ***p<0.001.

In controls and in CORT-nursed rats, induction of colitis caused a significant (P<0.001) decrease in body weight with respect to corresponding healthy rats (Fig. 1A).

Colitic control and CORT-nursed rats also showed a significant (P<0.001) decrease in food intake in comparison with healthy rats (Fig. 1B). In colitic CORT-nursed rats, the weight loss and reduced food intake were significantly (P<0.01) milder than those observed in colitic control rats (Fig. 1A and B).

### MPO activity

**Figure 2:**
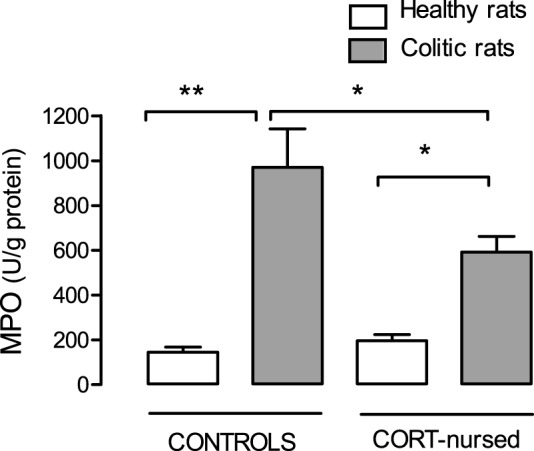
Effect of TNBS-colitis on colonic MPO activity in control and CORT-nursed rats. Four days after TNBS instillation, MPO activity was increased both in control and CORT-nursed rats with respect to the healthy condition. MPO levels in colitic CORT-nursed rats were significantly lower than in colitic controls. *p<0.05; **p<0.01.

In controls and in CORT-nursed rats, induction of colitis caused a significant (P<0.01 and P<0.05, respectively) increase in colonic mucosal MPO activity with respect to corresponding healthy rats. In colitic CORT-nursed rats, the increase in MPO activity was significantly (P<0.05) less than that observed in colitic control rats (Fig. 2).

### Histological evaluation

#### Macroscopic score

TNBS-induced colitis, both in control and CORT-nursed rats, caused the appearance of extensive ulceration and necrotic zones, tissue adhesions and, occasionally, diarrhoea. The macroscopic score was similar in both groups (Table 1).

**Table 1:** Intestinal length, macroscopic score and microscopic score in healthy and colitic control and CORT-nursed rats.

	Control rats		CORT-nursed rats	
	Healthy	Colitic	Healthy	Colitic
**Intestinal length (cm)**	22.50±0.33	18.38±0.34**	22.05±0.27	18.25±0.31***
**Macroscopic score (n.)**	0.0±0.0	6.66±0.35***	0.0±0.0	6.09±0.38***
**Microscopic score (n.)**	2.33±0.67	7.75±0.78 **	4.17±0.73	7.83±1.01 *

TNBS-induced colitis caused a similar variation in all the parameters considered in control and CORT-nursed rats. *p<0.05, **p<0.01, ***p<0.001 vs. own healthy group.

#### Microscopic score

The induction of colitis, both in control and CORT-nursed rats, resulted in submucosal and mucosal infiltration, increased numbers of inflammatory cells and blood vessel dilatation. The microscopic score was similar in both groups (Table 1).

#### Intestinal length

In controls and CORT-nursed rats, induction of colitis caused a significant and similar decrease in intestinal length with respect to corresponding healthy rats (Table 1).

### Plasma corticosterone levels and colonic mucosal GR expression

**Figure 3:**
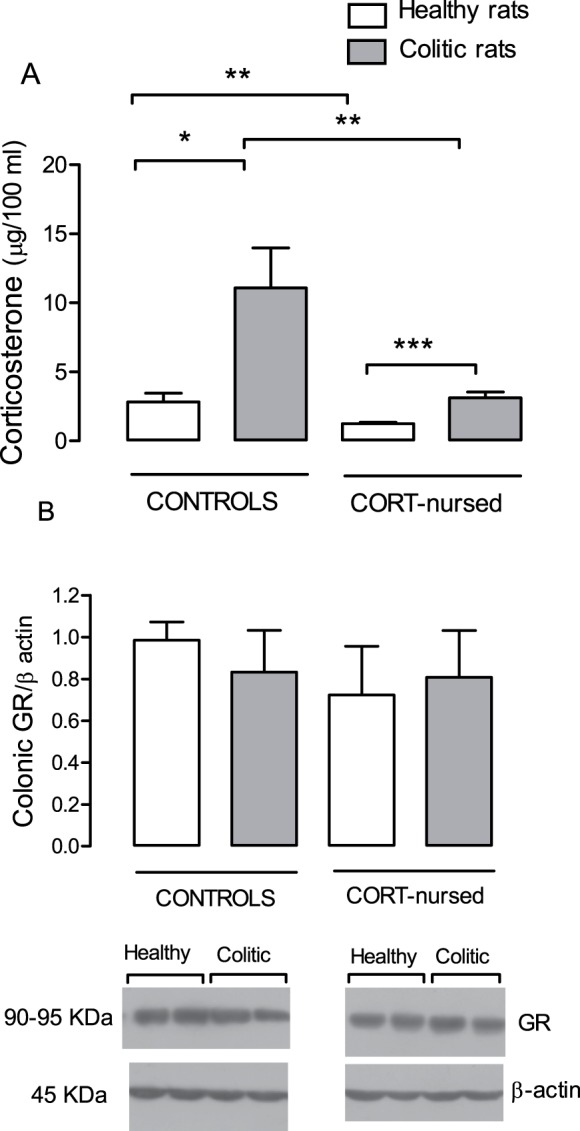
Effect of TNBS-colitis on plasma corticosterone and colonic mucosal GR expression. Four days after TNBS instillation, plasma corticosterone levels (**A**) were increased both in control and CORT-nursed rats with respect to the healthy condition. The increase in colitic CORT-nursed rats was significantly less than that in colitic controls. Moreover, healthy CORT-nursed rats showed plasma corticosterone levels lower than in healthy controls; *p<0.05; **p<0.01; ***p<0.001. Mucosal GR expression (**B**) was similar under all the experimental conditions. Representative Western blotting images were discussed above.

In healthy CORT-nursed rats, plasma corticosterone levels were significantly (P<0.01) lower than those observed in healthy controls (2.83±0.61 µg/100 ml). In controls and in CORT-nursed rats, induction of colitis caused a significant increase in plasma corticosterone with respect to corresponding healthy rats. In colitic CORT-nursed rats, the increase in corticosterone was significantly (P<0.001) less than that observed in colitic control rats (Fig. 3A).

Western blotting analysis revealed that GR were expressed in the colonic mucosa of control healthy and CORT-nursed healthy rats. Induction of colitis did not significantly modify the GR protein expression in either group (Fig. 3B).

### Mast cell number and density

**Figure 4:**
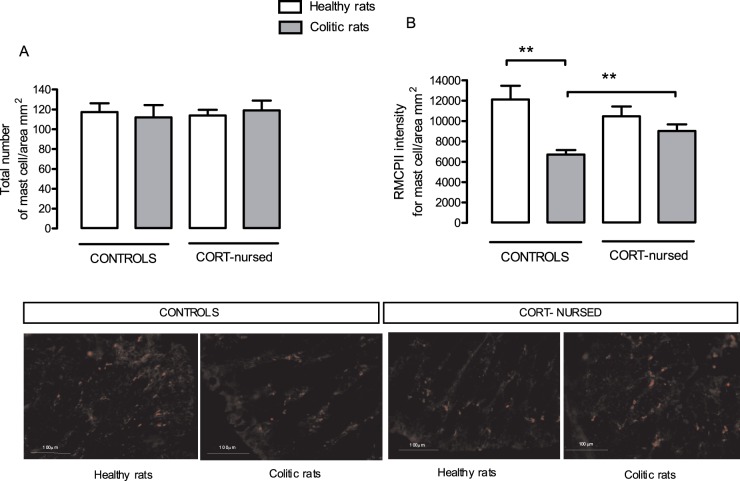
Effect of TNBS-colitis on colonic mucosal mast cell numbers and degranulation. The number of mucosal mast cells (**A**) did not change under any of the experimental conditions. The degranulation index (**B**) of mast cell activation, expressed as RMCPII intensity, was similar in healthy control and CORT-nursed rats, but significantly increased in colitic controls and unchanged in colitic CORT-nursed rats. **p<0.01. Representative images were discussed above.

Healthy control and CORT-nursed rats presented similar numbers of colonic mucosa mast cells, and the induction of colitis did not modify these numbers in either animal group (Fig. 4A).

In colitic control animals, a significant (P<0.01) decrease in mast cell intensity, corresponding to an increase in cell activity (degranulation), was observed; on the contrary, in colitic CORT-nursed rats mast cell intensity failed to be significantly modified, evidencing a reduced mast cell degranulation (Fig. 4B).

Representative histological samples of colonic sections from healthy and colitic controls, and from healthy and colitic CORT-nursed rats are reported in Fig. 4.

### Colonic CRF and CRH-1R immunostaining

**Figure 5:**
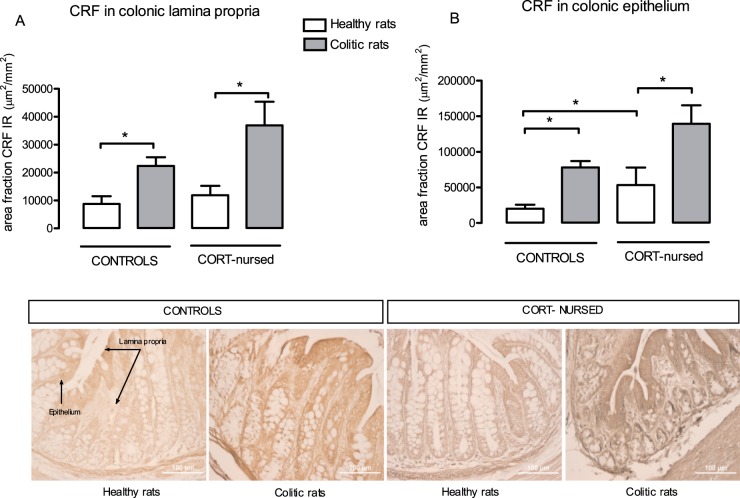
Effect of TNBS-colitis on colonic expression of CRF. Quantification (upper panels) and respective representative micrographs (lower panels) of CRF immunostaining in colonic lamina propria (**A**) and colonic epithelium (**B**) from healthy and colitic control and CORT-nursed rats. TNBS-induced colitis caused a significant increase in colonic CRF expression in both groups (**A** and **B**). In healthy rats, colonic epithelial CRF expression in CORT-nursed rats was higher than in controls (**B**). *p<0.05.

CRF immunoreactivity in colonic lamina propria of healthy control and CORT-nursed rats was similar, and higher in colonic epithelium of healthy CORT-nursed rats in comparison with healthy controls (Fig. 5 A, B). The induction of colitis significantly (P<0.05) increased CRF immunoreactivity in both animal groups, in lamina propria as well as in colonic epithelium (Fig. 5 A, B). Representative histological samples of colonic mucosal sections from healthy and colitic controls, and healthy and colitic CORT-nursed rats are reported in Fig. 5.

**Figure 6:**
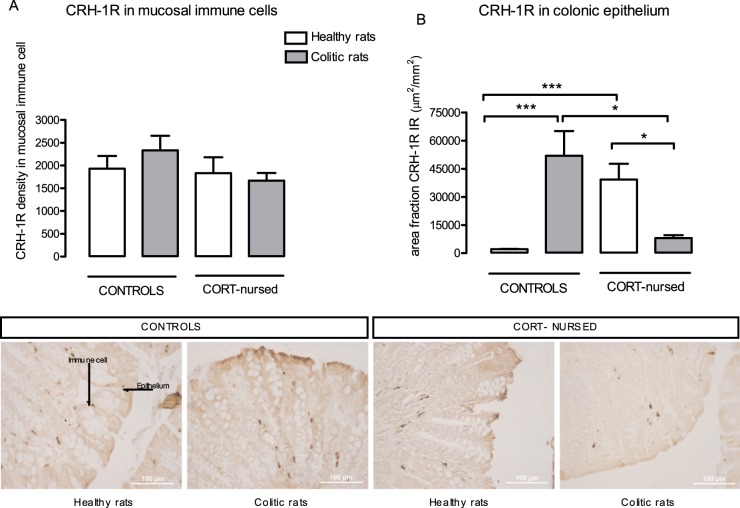
Effect of TNBS-colitis on colonic expression of CRH-1R. Quantification (upper panels) and respective representative micrographs (lower panels) of CRH-1R immunostaining in colonic immune cells (**A**) and colonic epithelium (**B**) from healthy and colitic control and CORT-nursed rats. TNBS-induced colitis, which didn't alter CRH-1R expression in mucosal immune cells in any of the groups (**A**), caused a significant change in colonic epithelium (**B**). In particular, colonic epithelial CRH-1R expression, which was higher in healthy CORT-nursed rats in comparison with healthy controls, was up-regulated in colitic controls and down-regulated in colitic CORT-nursed rats (**B**). *p<0.05; ***p<0.001.

Healthy control and CORT-nursed rats presented similar CRH-1R expression in colonic mucosal immune cells, which was not affected by the induction of colitis (Fig. 6A). In colonic epithelium, the CRH-1R immunoreactivity of healthy CORT-nursed rats was significantly (P<0.0001) increased with respect to healthy controls. After colitis induction, CRH-1R expression was significantly (P<0.05) reduced in CORT-nursed rats and significantly (P<0.0001) increased in controls with respect to corresponding healthy rats (Fig. 6B). Representative histological sections of colonic epithelium from both healthy and colitic control and CORT-nursed rats are reported in Fig. 6.

## Discussion

This is the first study indicating the beneficial effect of positive postnatal manipulation on the homeostasis of the gastrointestinal tract in the presence of an inflammatory disease. In fact, adult male rat progeny (CORT-nursed) of mothers whose drinking water was supplemented during lactation with moderate doses of corticosterone had a reduced vulnerability to TNBS-induced experimental colitis. Such a protective effect is revealed by improvements in several indices of the pathology (reductions in body weight and food intake, increased colonic MPO activity), and well correlated with a decrease in colonic mast cell degranulation. Conversely, colitic CORT-nursed rats, in comparison with colitic controls, did not show any variations in histological (macroscopic and microscopic) scores or in the typical shortening of intestinal length. It is important to note, however, that these parameters were evaluated in the early phase of the experimental colitis, corresponding to the maximum level of inflammation, when the healing process, responsible for recovery from the ulcers, oedema and restoration of a normal intestinal length, was probably not yet manifested. Thus, we cannot exclude that, in a late phase, recovery from these clinical signs could also be accelerated.

In the present study, we show that incremental maternal plasma levels of corticosterone that also increase hormone plasma levels in the newborns, mimicking a mild stress [26], promote, in the adult progeny, an adaptive phenotype more resistant to TNBS-induced colitis.

As reported here, healthy CORT-nursed rats are characterized by basal corticosterone plasma levels lower than those of healthy controls. This result confirms previous observations indicating that maternal corticosterone, during the neonatal period, induces a long-lasting hyporesponsive HPA axis in the adult progeny that represents a peculiar characteristic of the CORT-nursed model [27]. The persistent hyporeactivity of the HPA axis is also evident during colitis, as CORT-nursed adult rats had a reduced increase in plasma corticosterone under colonic inflammation with respect to colitic control rats. We can not exactly establish if and how the reduced HPA axis activity in adult CORT-nursed rats would be primarily involved in the reduced vulnerability to colitis we have observed. In previous studies we presented evidence how in the CORT-nursed rat model the HPA axis hyporeactivity and lower plasma corticosterone levels could play a crucial role in determining a phenotype characterized by reduced fearfulness in anxiogenic situations and resistance to ischemic neuronal damage [23], [38]. Certainly, the HPA axis is an important link between the brain and the gut immune system, and has a pivotal role in all the responses necessary to restore its homeostasis during stress, infections and inflammatory processes. Alterations in the responsiveness of the HPA axis are also recognized to be important components in the pathogenesis of several stress- and inflammatory-related gastrointestinal disorders [9], [10], [39]. In detrimental experimental models of neonatal manipulation, such as maternal deprivation (that, in general, produces outcomes opposite to those observed in the CORT-nursed model), a long-lasting HPA axis hyper-activation has been shown to be strictly associated with increased visceral hypersensitivity and intestinal inflammation, together with altered colonic barrier permeability [6], [7], thus suggesting a possible bidirectional role of HPA axis activity (i.e. ameliorating or worsening).

The dual role of the HPA axis in immune function may be strictly linked to the different endogenous levels of GCs released by the adrenal gland as a consequence of differential HPA axis reactivity. In fact, despite the well known immunosuppressive properties of GCs, thanks to which they have been used in the treatment of several inflammatory and immune diseases, it is known that they possess opposing effects that emphasize a bimodal regulation of immune responses [40], [41]. In this regard, the present data permit us to correlate a reduced vulnerability to an inflammatory stimulus with low plasma corticosterone levels. The biological effects of GCs are mediated by cytosolic GRs that, after translocation into the nucleus, interact with the promoter regions of different genes, controlling the transcription of immune-modulator factors. GRs are expressed in a variety of tissues, including the intestine, where they play a role in the control of the local inflammatory state [42], [43]. In CORT-nursed and control rats, we observed no changes in colonic mucosal GR expression, even if we cannot exclude the existence of a different receptor function (namely, receptor affinity) that, unfortunately, we could not examine because selective radioactive receptor ligands are no longer available. It is also possible that in CORT-nursed and control rats several post-transductional pathways could contribute to the activation/inhibition of transcriptional factors (i.e. NF-kB, AP-1) that differently modulate downstream signals and enhance an anti-inflammatory counter-regulatory mechanism, contributing to the reduced susceptibility to colitis in our experimental model.

Concerning the possible mechanism by which endogenous corticosterone induces its peripheral protective action on TNBS-colitis, the control of intestinal permeability could be suggested [44]. In particular, lower corticosterone plasma levels in CORT-nursed rats could promote the up-regulation of tight junction proteins and decrease colonic epithelial barrier function, ameliorating colitis. Some previous evidence tends to confirm this hypothesis [45], [46]. It is well known that high levels of glucocorticoids are associated with increased epithelial permeability after stress, in all regions of the gastrointestinal tract, which disappears after adrenalectomy or pharmacologic blockade of GR. In addition, experimental dexamethasone treatment has been shown to increase rat gastrointestinal permeability, mimicking the effects of stress [45], [46].

On the other hand, CORT-nursed rats have a new phenotypic profile that, in addition to the hyporesponsive HPA axis, has some other important peripheral characteristics that could be involved in the higher resistance to TNBS-induced colitis. We are referring to the intestinal CRF/CRH-1R system. CRF and CRF receptors, first described in the CNS, are also highly expressed in peripheral tissues. Peripheral CRF receptors contribute to stress-related colonic dysfunctions, being involved in changes in colonic secretion and permeability [30], and in particular, the activation of peripheral CRH-1R appears to mediate pro-inflammatory responses [47]. Previous studies indicate that the expression of the peripheral CRFergic system is altered during experimental colitis [43]. In this work we show, for the first time, that some long-lasting alterations in the colonic expression of both CRF and its receptor, in healthy and colitic CORT-nursed rats in comparison with controls, were induced by the beneficial hormonal stimulation during neonatal life. In particular, the expression of CRF and CRH-1R is increased in the colonic epithelium of healthy CORT-nursed rats in comparison with healthy controls, while it is unchanged in the lamina propria. Such a difference in the setting of the CRF system found in the adult CORT-nursed phenotype has to be associated, reasonably, with the increased (although moderate) neonatal exposure to corticosterone during lactation, but what is the mechanism underlying this event is not yet known. GCs are among the factors that influence the development of an effective gut barrier and epithelial integrity during postnatal life [47]. They play an important role in the maturation of digestive and absorptive functions, and stimulate morphogenesis in the small intestine and colon in humans [48]-[50] as well as in rodents [51], [52]. During the first twenty postnatal days, the responsiveness to GCs in the gut is increased [24] and GCs may influence stress-induced permeability changes in the colon [51]–[54]. In addition, GCs are able to influence the synthesis of CRF/CRH receptors [55] under stress conditions; thus, we suggest that the moderate increase in plasma corticosterone in the CORT-nursed pups during lactation, mimicking a mild stress, reprograms the peripheral epithelial CRF/CRH receptor system differently than in the controls. However, the long-lasting modification of colonic epithelial CRF and CRH-1 receptor expression observed in the CORT-nursed phenotype does not affect the homeostasis capacity of healthy CORT-nursed rats. Conversely, under TNBS colitis, an opposing modification of colonic CRH-1R expression corresponds to the up-regulation in colonic CRF expression, both in CORT-nursed and control rats. In particular, in CORT-nursed rats, unlike control rats [56], the induction of colitis caused the down-regulation of epithelial CRH-1R. The fact that the CORT-nursed phenotype, under basal conditions as well as under experimental colitis, shows a difference in expression of the peripheral CRFergic system, supports the involvement of this system in the reduced vulnerability to colitis observed in this model. CORT-nursed rats are more apt to counteract the homeostatic alteration induced by colitis through CRH-1R pathways, which are known to mediate pro-inflammatory processes [29], [57]. The down-regulation of CRH-1R in CORT-nursed rats could promote the reduced susceptibility to TNBS-colitis. The explanation why the differential regulation of CRH-1R expression can determine greater resistance to colitis in CORT-nursed rats, requires further investigations. Among the underlying mechanisms, an improvement in colonic epithelial function could be suggested. In fact, as colonic epithelial CRH-1R are known to mediate increased intestinal permeability [32], their down regulation, observed in colitic CORT-nursed rats, could result in a more efficacious barrier against luminal pro-inflammatory antigens. This proposal is in agreement with Soderholm et al., who, in functional studies with CRF antagonists, indicated that CRF is important for early life stress-induced changes in colonic epithelial function and suggested that its effects could be mediated by peripherally located receptors without characterizing their subclasses and location [58]. In this study we clarify that the subclass of CRF receptors mainly involved in modulating CRF-altered intestinal permeability is CRH-1R, located in the colonic epithelium.

The hypothesis that colonic epithelial functional improvement plays a pivotal role in the reduced susceptibility to colitis observed in CORT-nursed rats was put in evidence above when we discussed the role of their lower plasma corticosterone level, and it is also supported by another observation reported here. We are referring to the reduced colonic mast cell degranulation shown in colitic CORT-nursed rats, which may have a positive role in the preservation of the intestinal barrier. Mast cell activation increases gut macromolecular permeability following exposure to stress through the release of different mediators that enhance influx, altering trans-epithelial ion transport [33], [59]. The significant decrease in colonic mast cell degranulation observed in colitic CORT-nursed rats with respect to controls is in agreement with mast cell modulation of intestinal permeability, even if indirectly.

Altogether, these findings (the reduced plasma corticosterone levels, the down-regulation of colonic epithelial CRH-1R and the reduced colonic mast cell degranulation) contribute toward the hypothesis that changes in intestinal permeability are the basis for the reduced vulnerability to colitis observed in CORT-nursed rats. Certainly, because all neonatal manipulations are able to re-program numerous and different functions, the results reported in the present study cannot be considered as the only factors capable of enhancing resistance to colitis. Further studies are necessary in order to better understand the complex interactions between this neonatal manipulation and the reduced predisposition to developing intestinal pathologies exhibited by these animals.

However, we suggest that the CORT-nursed model represents an experimental model providing new insight into the field of gastrointestinal pathologies, and demonstrating that mild neonatal stress, similar to maternal care [60], induces long lasting physiological changes that make the adult better adapted to colonic inflammatory stress, probably through alterations in intestinal permeability.

In conclusion, the CORT-nursed model can be considered a useful tool to better explore other endogenous systems involved in individual susceptibility to colitis, in order to identify new therapeutic targets and approaches in the field of intestinal disorders.
